# Extra-Limites

**Published:** 1839

**Authors:** 


### EXTRA-LIMITES.

### Some Observations on the Treatment in Iritis. By Dr. *MacCabe*, Hastings.

Sir,—I have been led to offer the following observations from reading in the last number (61) of your Journal, a review of a work "On Diseases of the Eye," by Mr. Morgan.

My object is to suggest, that the treatment in iritis, when we have reasonable grounds to suspect a rheumatic or gouty diathesis, should essentially differ from that usually followed in pure iritis, and which I trust the following two cases may illustrate.

About eight years ago (I hope I may be allowed to say—better late than never), I was consulted by Sir F. Sykes, for some slight affection of the head which struck me at the time as being in some way connected with gout or rheumatism; in the progress of my attendance, he asked me had I heard of his celebrated sore eye? he said he had thirteen attacks of inflammation of the *same* eye in fifteen years; on each attack he had been blooded, generally to the amount of 60 to 80 ounces, with the usual grouting of leeches, blisters, cupping, lotions, &c. and confinement to a dark chamber from three to five months. I had the presumption to express a wish to break a lance (not a lancet) with this persevering antagonist, and he was good enough to say, that on the next attack, wherever he might be in England, he would give me the opportunity I wished for. In about four months from that period, on my way to Bath to visit a patient, I met Sir F. posting down to Hastings in performance of his promise, in the safe custody of his old foe.

On the next morning I visited my patient, and ordered a few ounces of blood to be taken from him by cupping, together with some other minor remedies evidently without any advantage, perhaps the reverse. On the following day I ordered dr. doses of turpentine with mucilage; cinnam. water and tr. of lavender, three times a day. On the morning after I was sent for, with a message, "that Sir F. was much worse," he had taken four doses of the medicine, but it was added with a *quite different complaint*, as the eye was nearly well. On my arrival I found my patient suffering from an obstruction of the bladder from which he was soon relieved by fomentations—but, as he informed me, after the third dose of the medicine, the pain had entirely ceased in the eye, and that which the day before, to use a homely expression, looked like a piece of bullock's liver, had a brownish, I may say faded, appearance.

One or two draughts a day perfected the cure, and I understand that on a subsequent slight attack, a recurrence to the same medicine was followed by similar results.*

*Case* 2.—About three years since, Mr. C——d, son of Sir C. C——d, put himself under my direction for a severe inflammation of the eye; this was the second or third attack; the last of which left a slight speck on the cornea. Here also I had good cause to suspect a gouty taint in the constitution. Sir

* I recollect that about the period alluded to, I accompanied Sir Francis to London and got him to consult the Senior Editor of this Journal for some complaint of his digestive organs.

C. happening to be in London at the time his son was under my care, he described the symptoms to Mr. Alexander, who had treated Mr. C. on the last occasion. Mr. A. suggested a couple dozen leeches to the forehead, which out of respect to that eminent occulist, I reluctantly acceded to; the consequence was evidently injurious, even to the datient's feelings; his sufferings being increased. I then ordered the turpentine as in the last case, with immediate relief of all the symptoms, and in eight days he was able to resume his usual occupations and amusements. In this instance no metastasis took place.

---

### SIR JAMES MURRAY'S RECLAMATION.

*To the Editors of the Medico-Chirurgical Review.*

33, *Piccadilly, London, Aug.* 24*th*, 1839.

GENTLEMEN,—Last winter I apprized you that in future you could obtain my solution of magnesia from a *then* agent of ours in London. That person has since set up for himself, and has garbled the eulogium which you wrote in your Medico-Chirurgical Review; and has applied your testimonial to an imitation of his own, not in existence when you published your favourable opinion of the genuine article.

If characters intended for one person or thing can be thus transferred to the purposes of others, then all confidence would be at an end, particularly in matters connected with medicine, health, and life; and the merit and credit of all discoveries and improvements would be alienated in open day. As a friend of fair dealing I beg of you to favour me with your opinion of such conduct.

I remain, Sirs, yours sincerely,

JAMES MURRAY.

N.B.—We have continued to use and prescribe Sir James Murray's solution of magnesia, and the character given of it in our Journal, was, of course, applied to the original.

---

### ABSTRACT OF A PAPER READ AT THE MEDICAL SECTION, BRITISH ASSOCIATION, BIRMINGHAM, 28th AUG. 1836. By Sir *James Murray*.

" MR. PRESIDENT,—The seventh Report of this great Association contains a paper which I had the honour of submitting two years since at Liverpool: I there detailed the appearances which I discovered upon dissecting two persons who had died of excruciating local pains or neuralgia, and where I found the nervous membranes of the diseased parts studded over with a frost work of minute or microscopic crystals, presenting a large surface of irritation. Upon analysing these crystals, and also the crusts of scald-head and lepra, and the discharges from old ulcers, I found a large proportion of urinary salts, and particularly uric and compounds. In the same paper many proofs were adduced, demonstrating that many other complaints originate from and forming in the stomach, and eventually pervading the system, where their acrimonious deposits produce diseased action, and thus frequently become the causes of gout, gravel, and even of febrile excitations and nervous affections. I further pointed out that such acerbities produce or aggravate those thousand forms of dyspepsia which are generally accompanied by the presence of acrid matter in the alimentary canal. Lastly I proved, that thirty years' experience had left no doubt of the immense superiority of fluid magnesia, not only for correcting those acids evolving in the stomach, but also for dissolving and washing away those saline and acidulous impregnations with which the solids and fluids are frequently imbued.

Soda and potass are injurious in such cases, because most of the noxious saline depositions contain *urates* of those very *alkalies.* Solid magnesia is objectionable, because it cannot enter the circulation, nor follow and neutralize acidities in distant organs. Besides this, it settles in concretions itself, as I every year observe during my observations as Inspector of Anatomy.

When concluding my paper at Liverpool, I was requested by many members to describe the fluid magnesia at a future meeting. I now present it before you, and trust when you consider that the crude earth is a source of injury to almost every infant, you will not accuse me of undue partiality to this *first offspring of my earliest labours and studies,* or that I attach more importance to its extension than its merits deserve.

You observe the fluid is colourless and almost tasteless, that infants and infirm persons can take it without compulsion or disgust, and it is well known that it will remain upon irritable stomachs which reject all other liquids. The copious extrication of its carbonic acid hinders the undue evolution or deposition of phosphates or white sand.

By boiling the clear solution in this tube you can prove three things :—that no degree of animal heat will render the magnesia solid again in the body, in fact, it does not subside under a temperature of 212° :—that I have brought the fluid to such perfect condensation *(aided by my late brother during his residence in London)* as to contain about a scruple of the bi-carbonate of magnesia in the ounce of distilled water, and that by a series of processes it is cleared from gypsum, silex, lime and other impurities contained in common and calcined magnesia from the nature of their manufacture,

Gentlemen, after fair trials, you will admit that rendering the carbonate of magnesia soluble, and completely pellucid in *distilled water,* and administering it in the fluid form, is a more important improvement than people at first sight might suppose. As I elsewhere said, it is not disagreeable to take, nor liable to the subsequent consequences of swallowing the crude drug in its mechanical form of paste or whitewash.

This is a manifest advantage in the mode of exhibiting a medicine of *daily and hourly employment in every nursery and sick-room.* You know that the solid earth and its mixtures, such as Gregory's powders, are in the hands of all persons, medical or otherwise, and are mixed up for every age and every ailment. In my essay on *Dilution* (Longman, 1829) I pointed out the danger of giving crude magnesia, proofs of which had occurred to Dr. Mc Cluny of Belfast and myself in 1809.

The discovery of these dangers led me to endeavour to render available a known principle of nature, to new purposes in medicine, by novel processes of art. For this purpose I spared no expense in procuring engines capable of solidifying carbonic acid gas in contact with magnesia, so as to hold that earth dissolved in distilled water. You will observe how entirely this single and pure solution differs from those decompositions spoken of in old dispensatories, as adopted by recent fabricators, that is *mixing sulphate of magnesia with carbonate of soda,* the resulting portion contains *Glauber salt,* a crude dose for tender females or infantile disorders ! ! It is monstrous to hear of and see the conduct of these numerous substitutors of one medicine for another.

That such persons can have no excuse on the score of ignorance of the original article, you will admit with me, when I recount the pains taken in conjunction with my late two brothers, respected medical men, to make this preparation known to the profession in every part of the world. Dr. Hope, of Edinburgh, at my request, shewed it to his class in 1813—14—15. Professor Duncan recommended it in his dispensatory in 1819—making honorable mention of my name as the inventor. In 1816 I had printed a clear account of it in the Newry Magazine, edited by the present James Stuart, Esq. LL.D. Some years before, Dr. Richardson, F.T.D. honoured me by his celebrated letter, in which he described his former sufferings from gravel, and his perfect cure by this solution. Many of your present eminent members gave me credit for this useful improvement during twenty years in their lectures, such as Professors *Barker* of Dublin University, *Upjohn* of the College of Surgeons, *Moryson* of Glasgow, and the late *Turner* of London and M'Donnell of Belfast, can assure you that he found it to dissolve urinary calculi in 1812. Dr. James Johnson of London, physician to the late king who was present at the reading of my paper at Liverpool in 1827, and also at the London and Westminster Medical Societies, sent to me to Dublin for a supply of the fluid magnesia for his own use, and recorded in his *Medico-Chirurgical Review*, for April last, *the very great benefit he derived from it*, adding a request (since suppressed in the hand bills of an imitator) as follows: "*We hope Sir James Murray, the discoverer of the process for preparing this medicine, will take the trouble to make it more generally accessible to the public in this Metropolis, there being only one or two authorised agents here.*" This very agent garbled this eulogium of Dr. Johnson, struck out my name, and applies the testimonial in open day to a compound of his own, substituted by himself in London, and *commenced three months after Dr. Johnson's recommendation was written!!!* Dr. Johnson, from his love of fair dealing, will, sooner or later convict this pretender and settle the point, whether he recommended the original preparation invented by Sir James Murray, or the substitute produced long afterwards by an agent, an imitator, Mr. Dinneford.

The original handbills of this Dinneford, printed by his own order, and circulated by him in May last, when he was selling "*Murray's Solution,*" may be seen at 33, Piccadilly, in which he styles himself "SOLE AGENT FOR THE UNITED KINGDOM." In June he reprints the same hand bills, *almost verbatim*, (only *altering the names*) and appropriates to his own substitute the letter of Dr. Cumins, and the references to Professors Duncan, Hope, Turner, Moryson, Barker, and Sir Humphrey Davy, which were entrusted to him by his principal, and recorded many years before his imitation was thought of, even by himself.

Gentlemen, Dr. Thompson in his evidence before the House of Commons, proved the danger of vending one preparation under the character and recommendations long awarded to another.

I had resolved not to mingle in either *chemical* or *commercial operations*, but the demise of my late relatives, to whom I had consigned these matters, and the consequent breaking up of their laboratory obliged me to come forward, and protect the preparation from the open piracy of an agent commencing for himself, in June, 1839, long after every improvement was perfected by my family in London or elsewhere.

Gentlemen,—The time to decide disputes about claims is when the *parties are alive*, and witnesses present; fortunately many distinguished members of your body are now here from Dublin, Belfast, Edinburgh, and Glasgow, who have often recorded their high approval of my invention, and can now prove to you that I spent a long life and a fortune in bringing to perfection a medicine of daily and hourly advantage to old and young. As a member of your body, I claim your protection of it, and that you will secure the merit and reward only to him to whom they are justly and undoubtedly due."

The Section expressed satisfaction upon seeing the analysis of the fluid magnesia, and passed an unanimous vote of thanks to Sir J. Murray.

### Dr. Macfarlane's Case.

*(To the Editors of the Medico-Chirurgical Review.)*

*Leeds, May* 27, 1839.

Gentlemen,—If you consider the following case of sufficient interest to merit a place in your valuable journal, I shall feel obliged by its insertion.

Feb. 14, I was requested to see Martha Thorpe, æt. 28; complained of tightness in the chest; wheezing, troublesome cough; headache, which was much aggravated by cough; eyelids slightly œdematous; no flushing of face; legs slightly œdematous; P. natural; had been in this state nearly a week, during the previous night had a good deal of pain in the abdomen, but was quite free from any pain there when I visited her; B. natural; thirsty; she quickened about 5 weeks previous. Ordered her a smart purgative, and in the evening a mixture for her cough, containing squill with a few drops of tr. opii. 15th. Feels a great deal better; slept well; cough not very troublesome; b. freely purged; no head-ache, thirst, nor pain in abdomen. 16th. I was hastily summoned about 8 a.m., she had had a great deal of pain in abdomen on the evening of the 15th, attributed to eating rich cake to tea, which continued until 12 p.m. and then ceased, and she was easy and slept until her husband got up to go to work, when she complained being very cold. Before I was sent for she was seized with an epileptic fit; I found her just becoming sensible. Complained of her head being confused; I observed some bloody saliva on the pillow, which told me the nature of the fit; another immediately followed. I bled her to the amount of 20 oz. and before 9 o'clock she had 5 fits. On examining, per vaginam, there was no indication of labour. The fits still continued. Before eleven, a.m. I bled her three times to the amount of nearly 50 oz. ordered the hair to be cut off, vinegar and water applied, blister betwixt shoulders, sinapisms twice to the feet, soap suds to be injected per anum every hour, and a strong infusion of senna with salts to be given, if she could swallow. In the afternoon the fits were less frequent but still as violent; the pulse was so much reduced that I did not think it adviseable to bleed any more. Ordered a mixture containing ant. tart. gr. vj. in aq. font. ℥vj. c. c. ij., om. hor. I visited her eight or nine times during the day, and there were no indications of labour; her tongue was dreadfully cut and swelled, and from the quantity of blood that flowed from the mouth the attendants thought a blood-vessel had broken. The left middle incisor tooth was knocked out, its fellow and the dens caninus is quite loose, and she has since pushed them out with her fingers; is quite insensible. 17th. 3 a.m. I visited her; had no fit since half-past 12, until I was in the room; had taken 4oz. of the mixture; the uterus seemed lower in the pelvis but no dilatation of the os uteri. Contin. mixt. et addit gum camph. ℈j. 8 a.m. No alteration. 10. a.m. No alteration. 12 a.m. On examination found the os uteri more soft and yielding, and could introduce the tip of forefinger into it. I desired to be sent for if there was any vaginal discharge or any pains; about half-past one I was sent for, and found the membranes protruding; on rupturing them I found the fœtal head in the pelvis, and the pains continued and expelled the fœtus, immediately followed by the placenta; the uterus contracted and the patient continued quite insensible, but seemed to assist the pains by bearing down. No fit during labour nor since 12 a.m. until 18th, about 3 p.m., which was the last; the bowels were moved by enemata; urine passed involuntarily. 18th. The catheter was introduced but only a few drops of urine flowed; lochial discharge scanty; b. moved twice; urine passed involuntarily; continues insensible; p. 120 weak; contin. mixt. 19th. Rather sensible this morning, answers questions; cough very troublesome; the swelling of tongue rather diminished; p. quick and full. Vespere. Became rather incoherent; heat of surface; headache ; p. sharper ; eyes rather bright. Ordered hirud. vj. tempor, and ant. tart. gr. vj., aq. ℥vj., c. c. j., maj. q. 2da hor., cold to the head. 20th. Passed a restless night, but not delirious ; cough troublesome ; can talk better and answers questions ; bowels not moved ; headache ; p. quick and feeble. Ant. tart. gr. iv., muc. g. acac. ℥j., nit. pot. ʒj., aq. ℥vij., c. c. ij., mag. 3tia q.q.h. Infus. sennæ c̄ mag. s. statim. Takes lemonade, tea, and sago greedily ; vespere ; complains of tongue ; p. 120 ; bowels acted and urine also passed involuntarily. 21st. Has passed a tolerable night ; headache ; pain in abdomen ; cough troublesome ; expectorates viscid phlegm ; thirst ; slight heat of surface ; swelling of tongue diminishes ; urine passed in bed ; introduced catheter and drew off 7oz. turbid urine ; slight lochial discharge. Vespere—p. quick and feeble ; pain of abdomen easier, headache ; asked for pudding, and had a little rice pudding and steeped bread for dinner, being the first solid food since the evening of the 15th ; ordered the lemonade to be discontinued, it having produced griping ; the cold not to be constantly applied to the head ; tried to pass urine but could not ; drew off 6oz. by catheter ; contin. mixt. 22nd. Improving gradually ; had the bed made to-day ; ordered beef-tea ; bowels moved and urine passed naturally ; ordered the mixt. to be continued with acid hydrocyan. min. xvi. 23rd. Improving a little. 24th. Ordered squill and tr. opii. in mixt., to drink linseed tea with extract of liquorice at pleasure. 25th, 26th, and 27th. Improving, cannot remember that I visited her on 14th and 15th ult. 28th. Improving. March 1st. Last night she was taken out of bed and carried down stairs and stood upon a stone floor for a minute or two ; about midnight severe pain and swelling of right leg came on, and prevented sleep. On examination found the leg swelled from the toes to the knee, but not much swelling of the thigh ; pain on pressure in the groin and along the course of the absorbents ; cough rather easier ; not much heat of surface ; b. regular ; p. quick and feeble ; ordered hirud. vj. to be applied to the groin, calomel gr. ix., pulv. ipec. and opii, ʒss. divid. in vj. c. j. bis die. ℞. Adip. suis. ℥j. ung. hyd. fort. ℥ss. extr. bellad. ʒj. ft. ungt. vas. absor. sup. applicand. noc. maneque. The limb to be enveloped in flannel ; beef-tea ad libit, and nourishing diet. 2nd. Rather better night ; very little pain of leg or groin on pressure ; feels sickly ; no thirst nor head-ache ; perspired a little during the night ; rep. hirud, inguin., contin. med. et alia. Vespere—very little pain on pressure ; there was tendency to syncope and the bleeding was stopped and a little wine given ; been griped and had two motions from mercury ; feels sickly ; discontinued the powders and ordered tr. opii gr. xxxv. h.s., to have nourishment frequently during the night. 3rd. Has had a pretty good night ; no pain of leg and only in the groin when pressed upon ; glands slightly enlarged ; feels much better this morning ; tendency to syncope not so frequent ; cough still troublesome ; can make a little use of leg ; the ointment to be continued, and flannel bandage ; p. 116 ; tongue furred ; no thirst ; swelling rather subsiding, except about the ancle ; ordered decoct. cinch. ter die, nourishing but not stimulating diet, tr. opii. gr. xj. h. s. 4th. Pretty good night, improving in every respect. 5th. Still improving. 6th. Has had a good deal of griping and several motions during the night ; ordered tr. opii grs. xxx. 7th. Had pain in the back of thigh, but no pain in the inside ; swelling considerably abated ; ordered frictions with olive-oil and laudanum. 8th. Has had griping pain in the bowels ; appetite not quite so good ; feels sickly from griping ; ordered the ointment to be discontinued, and a little wine to be given. 9th. Continues improving. It is needless reporting any farther ; the patient slowly kept improving ; the leg is yet swelled and weak after exertion. I have recommended Harrogate—to try warm bathing ; she can, however, go about her affairs. It was the opinion of her attendants that she had in all nearly 40 fits, and she was not delivered until 30 hours after the supervention of the first fit. I never witnessed epilepsy in so severe a form, I have seen it very severe in drunkards, (and also in a previous case of puerperal convulsions, which, however, did not come on until 3 hours after delivery, and was treated in the same way, the woman recovered and has had a child since), but not at all so severe as in Thorpe's. I attribute her recovery entirely to the powerful antiphlogistic treatment adopted, and not interfering with the expulsion of the uterine contents. The principal peculiarity was the convulsions attacking at so early a period of pregnancy, and preceded by a severe catarrh.

I intended offering a few remarks, but a fear of taking up too much room warns we to proceed no further. I have merely stated facts as they occurred and leave others to make their own remarks on the propriety of the treatment adopted, &c.

I have to acknowledge myself considerably indebted to your review of Dr. Collins' work on midwifery for the plan of treatment employed, and I bear my humble testimony to the value of that portion particularly which treats of convulsions.

I remain, Gentlemen, yours, very respectfully,
JOHN MACFARLANE.

23, *Wellington Street.*

---

### Ptisane de Feltz.

*Communicated by Doctor Fergusson of Windsor.*

Conceiving it always of importance for the medical profession here to be made acquainted with the proceedings of their professional brethren on the other side of the Atlantic, I herewith send for insertion, if you see fit, in the forthcoming number of your Quarterly Review, the substance of a communication, dated January, 1838, from my friend Dr. Lynch O'Connor, addressed to the Medical Society of Trinidad, on the efficacy of, to us, a new remedy in some of the anomalous forms of pseudo syphilis, which there had either been mistaken for lepra, and abandoned as incurable; or as true syphilis, and subjected without benefit to severe and protracted courses of mercury. The remedy is known in France under the name of the Ptisane de Feltz. Dr. O'Connor has seen it used with great success in the Parisian hospitals, where it was prepared according to the following formula of Monsieur Cullerier, at the Hôtel Dieu.

℞ Sarsaparillæ contusæ ℥ii—Schthzveull. ℥ss

Sulphuret. antimonii ℥iv—Aquæ Bullient. O. vi.

to be slowly boiled down to three pints, and a pint to be drank daily. The following cases are given in proof of its powers.

1. Lewis, aged twenty years, till within two years a healthy negro, was placed by his master under my care on the 18th of April, 1831, at which time he was suffering from ozœna—emaciation—hectic fever—nose apparently excavated and tumefied—upper lip tumid and inflamed—soft palate sloughing, with a covering of viscid mucus on the edges, and a blush of dark inflammation extending over the roof of the mouth—voice hoarse— hearing impaired—larynx evidently affected, as the slightest pressure on the thyroid cartilage produced pain, and was succeeded by sanguinolent muco-purulent expectoration, and the deglutition so difficult that he could barely satisfy the cravings of nature. Having ascertained that he had previously, under the care of two professional gentleman, given the fairest trials to sarsaparilla and mercury, I considered the case as hopeless, from having met in practice five or six cases in this colony of a similar nature, all of which terminated fatally; but recollecting the beneficial effects I had witnessed from the Ptisane de Feltz, at the Hôtel Dieu, I determined on giving it a trial, with only the addition of an injection into the nose of 40 min. nitrous acid to a pint of water. After two months steady perseverance, he was restored to perfect health. He was personally inspected by me last Saturday, when I found the only remains of disease to be a nasal intonation of voice, and, from the destruction of the soft palate and uvula, an occasional difficulty of swallowing liquids, which returned through the nostrils, unless he drank slowly and cautiously. The fistula of the palate was completely obliterated; and it is worthy of remark, that neither he nor his parents ever had any symptoms of yaws or venereal disease.

2. Mr. George Reed, a native of England, aged 48, and a notorious drunkard, had been affected for 18 months with extensive cutaneous ulcerations, of a florid hue with elevated edges, and deep-seated pains in the bones. As in the former case, mercury and sarsaparilla had been used to the utmost extent in vain. He denied having ever had any venereal infection, and his wife, with whom he cohabited, was perfectly healthy. The same remedy was administered, with the addition of opium to allay the nocturnal pains. I first visited him on the first of July, 1831, and although he drank a pint of new rum daily, he was perfectly cured on the 23d of August following. He continued in his abominable habits for five years afterwards, without any relapse, and was ultimately drowned in a fit of drunkenness.

3. A professional gentleman, aged 30, in the month of November, 1832, placed himself under my care and that of Dr. Pardy, in the last stage, apparently, of ulcerated nodes and hectic fever, having been bedridden upwards of five months. It appeared, that two years previously he had contracted, in London, a primary excavated ulcer, which was easily cured by a moderate course of mercury; and that about 18 months ago, he began to suffer from nocturnal pains, which were followed by deep-seated extensive ulcers in the forehead and extremities, for which mercury, sarsaparilla, and the mineral acids were used in vain. He was covered with ulcerated nodes—reduced to a skeleton, through excessive pain and want of sleep, which had induced insanity. He was made to drink the Ptisane de Feltz daily, with a full dose of laudanum at bed-time, and, in little more than two months, a perfect cure, without the assistance of any other medicine than a very small dose of the oxymuriate of mercury during the first month of the disease. He never relapsed, and is now residing in London.

4. A Creole negro, aged 26, healthy from his birth, after amputation of the leg, from an accident, was attacked with all the symptoms of the first case in so aggravated a degree, that the nasal and palatal bones had exfoliated; although, so rare is syphilis in the West Indies, neither he nor any of his progenitors could be traced to have been affected either with that or other constitutional malady for two generations. It baffled every attempt at cure, or even alleviation, with the ordinary and most approved remedies, and was perfectly cured in two months, under precisely the same treatment as the last. I personally inspected him last Saturday, and found him in good health, although much disfigured.

To the above may be added two female apprentices of the magistrate, exhibiting striking cases of this protean malady, in a desperate form, resisting all remedy; but at last obtaining perfect ease through the formula I have been recommending—as also the child of a respectable inhabitant, and the captain of a merchant brig, and others of our European colonists, who have unquestionably been saved from the most malignant cutaneous disease through its operation.

---

### LACERATION OF THE PERINEUM CURED BY OPERATION. By *Robert Davidson*, Esq.

THE restoration of the perineum to its natural state, after having been lacerated in labour, is unquestionably one of the greatest triumphs of surgery. In the following case, which we extract from the Lancet of May 4th, 1839, the patient was restored to society by the operative skill of Mr. Davidson, and the medical tact of Dr. Henry Davies.* We have learned, from the best authority, that the lady is in good health, and moves about with that cheerfulness and elasticity which youth and high spirits alone can bestow.

"The subject of this case was a lady about 29 years of age; the accident took place in her second accouchement. The laceration of the perineum was complete from the vulva to the anus, and extended from half to three quarters of an inch into the recto-vaginal septum; the consequence of this was an involuntary discharge of fæces, to the great distress of the patient and her friends. Feeling much anxiety about the case, from the knowledge of the want of success which had so frequently attended the operation, I referred to the cases recorded on the subject.

The able memoir, by Baron Roux, fell under my notice, and as the result, in several cases, had proved so very satisfactory, I determined on adopting the plan he pursued,—that of the quilled suture,—with a little variation, which I will presently describe.

Having explained my views to Dr. Henry Davies, whom I had the pleasure to meet in consultation, he perfectly agreed with me on the method I proposed to adopt; and although only twelve days had elapsed since the accident, the patient was so anxious to have the operation performed, that I did not hesitate to do it, having had the bowels previously well opened by means of a purgative and enemas. On the 6th November, in company with Dr. Henry Davies, I performed the operation in the following manner:—I passed deeply a strong double ligature, by means of a common curved needle, close by the edge of the rectum, and another rather more than half an inch apart from the first, towards the vagina, after which I pared the edges of the wound, which I had not previously done, that I might not be annoyed by the oozing of blood, so as to be enabled to place the ligatures more accurately. The ligatures being introduced, I employed, as cylinders, two pieces of elastic gum catheter, about an inch and a half in length, one of which was placed in the loops which the double ligatures formed on one side, and the other between their separate ends, tying them firmly upon the cylinders. Baron Roux found in his cases that the use of the quilled suture caused an eversion of the edges of the wound; to remedy this he had recourse to several small sutures, at different points, between the different ligâtures. To effect the same object, and also with a view of keeping the divided parts more closely and firmly in contact, I adopted the following plan, the materials for which I had prepared previous to the operation. I armed a curved needle with a piece of narrow tape, four inches long, having a knot at one end; this was passed down each end of both cylinders about half an inch, and brought outwards, the tape being prevented slipping through by the knot; the tapes then were thus placed in such a position as to be intermediate to the ligatures; this being done I turned the cylinders gently towards the edge of the wound, and tied the corresponding tapes over it, which I think rendered it much more solid than any number of small ligatures could have done. After the completion of the operation there was so little tension of the parts that we thought it quite unnecessary to make the lateral incisions so strongly recommended by M. Dieffenbach. The wound was dressed with simple dressing, and a roller applied to the knees to guard against involuntary motion during sleep. The mistura cretæ, with the liquor opii sedativus, was given daily to restrain the action of the

---

* We wish that this excellent physician would publish some of the results of his long and successful practice; it would make an admirable volume for the practitioner as well as the student.

bowels, and severe diet ordered, to such an extent that during seventeen days nothing was allowed but gruel, and that in small quantities, with occasionally a little hard biscuit. This treatment, however, the lady endured with the greatest patience and fortitude.

The urine was drawn off night and morning, which, as she lay on her side, with the thighs flexed on the body, was frequently attendant with no small difficulty.

No untoward symptoms occurred, and on the seventh morning the ligatures were removed, when it was found that firm union had taken place along the whole extent of the wound, with the exception of a small fistulous opening near the edge of the sphincter, having no communication with the rectum, and producing no inconvenience to the patient. After nine or ten days the *urine* was passed without assistance, the parts being guarded by a pledget of linen; the small fistulous opening was touched night and morning with tinct. cantharidis, and occasionally with argenti nitras, with the best possible effect.

On the seventeenth day no evacuation from the bowels having taken place, we administered a purgative draught and laxative enemas, which producing no effect, were repeated; but so impacted had the fæcal matter become that there was much difficulty in accomplishing our object, so that we were obliged to have recourse to manual assistance. We were not without our fears as to the effect of the action of the bowels on the newly united parts, but the union was so firm as to sustain no injury. When regular action was established, she was allowed gradually to move about, and at the end of six or seven weeks she resumed her usual habits. During the whole time the infant was applied to the breasts at intervals, sufficient to relieve them, and keep up the lacteal secretion, as she was anxious to continue nursing."

### HEIGHAM HOUSE, NORWICH.

### A RETREAT FOR INVALIDS AFFLICTED WITH MENTAL DISORDER,

*Under the Superintendance of the following Proprietors:—*

WARNER WRIGHT, M.D., Fellow of the Royal College of Physicians in London; Senior Physician to the Norfolk and Norwich Hospital; Physician to the Norfolk County Lunatic Asylum, and to the Norwich Bethel.

WILLIAM DALRYMPLE, Esq. Honorary Consulting Surgeon to the Norfolk and Norwich Hospital; Surgeon to the Norfolk County Lunatic Asylum, and to the Norwich Bethel.

JOHN GREEN CROSSE, M.D., F.R.S., Senior Surgeon to the Norfolk and Norwich Hospital, &c.

This Establishment, which has existed above half a century, was rebuilt some years since upon an enlarged scale, so as to afford superior accommodation to the highest class of patients; it is situated in one of the most retired, healthy, and cheerful spots in the kingdom. The building is replete with every modern improvement for the comfort and care of invalids; and the lawn, plantations, and walks, extend over twelve acres of ground, all appropriated to the use and recreation of the inmates.

There is a resident master and matron, and daily professional attendance is given. Attendants accustomed to the care of invalids can be sent to any part of Great Britain.

Applications to any of the Proprietors will receive immediate attention; further particulars may also be obtained in London, of Mr. Battley, White Cross Street, Cripplegate; or Mr. John Dalrymple, 6, Holles Street, Cavendish Square.

*Remarks.*—We have great pleasure in directing our readers' attention to this Establishment. The names of the proprietors are the highest possible recommendation.

---

### Abstracts of remarkable or interesting Cases, read at the Medical Section of the British Association, 26th August, 1839.

The following remarkable cases, the particulars of which we shall hereafter communicate, were read by our friend Sir David Dickson.

Case of rupture of the duodenum in four places.

Case of *ileus* from strangulation, producing sphacelation of the cæcum, which was so distended as to occupy the situation of the transverse colon.

Case of intermittent *coma* (exhibiting, though the cause was progressive and organic, remarkable alternations of torpor and excitement) from diseased brain, and rupture of the anterior artery of the cerebellum.

Case of *arachnitis,* exhibiting, besides the usual cerebral lesions, very extensive formation of little bodies of semi-cartilaginous firmness in the sub-serous tissues of the diaphragm, stomach, spleen, mesocolon, &c.

Case of sudden *coma* in a convalescent (from abscess) from extravasation of blood, in which large tubercles were found attached to the anterior extremity of the right olfactory nerve, and to the optic thalamus. And also (as in another case of phthisis) with very extensive crops of tubercles resembling pustules, in the subserous tissues of the abdominal cavity.

Case of *phthisis* with the *foramen ovale open,* in which the patient had completed the allotted period of service and attained the rank of serjeant, without cyanosis, or (until *latterly*) suffering any particular inconvenience.

Case of *phlegmonous erysipelas* of extraordinary rapidity and extent. (The man pulled out to the sound on the 4th, and died in the night of the 7th—8th August.

Case of *constant vomiting* from *chronic pancreastitis,* and scirrhus of the pylorus, and ulceration of the duodenum.

Case of *peritonitis* and *scirrhousca,* exhibiting (besides considerable effusion) the extensive deposition of an icthoid substance of simicartilaginous firmness in the subserous cellular tissue of the intestines. It was of a dull white, yet glistening fishlike appearance, fibriform, and from half an inch to upwards of an inch in thickness in some places, and had separated the peritoneal from the subjacent coats so completely, that even yards of the latter might be drawn out from the former with great facility.

---

### Birmingham Royal School of Medicine and Surgery.

The anniversary meeting of this institution took place on Wednesday. The Theatre was crowded to excess, and many distinguished visitors were unable to obtain admittance. The Town Hall had been secured for the occasion, but the arrangements for the reception of the British Association prevented its use. Dr. Johnstone, the President, was called to the chair, having on his right hand the Earl of Dartmouth, the Rev. Dr. Buckland, and the Rev. J. P. Lee; and on his left Lord Viscount Lifford, Sir J. Mordaunt, Bart. M.P., the Hon. Mr. Hewitt, C. H. Bracebridge, Esq., &c.—Letters apologizing for absence were received from the Marquis of Northampton, Earls Stamford, Bradford, and Mountnorris; Lords Bagot, Calthorpe, Lyttelton, and Wrottesley; Sir E. Wilmot, Bart., Sir

A. Cooper, Bart., E. J. Shirley, Esq. M.P., Wm. S. Dugdale, Esq. M.P., &c. &c.—After a short address from the President, the following Report was read by the Rev. Mr. Clark, Rector of Northfield :—

"The Council of the Royal School of Medicine and Surgery, in presenting to the Governors on this their eleventh anniversary their report of the progress and proceedings of the Institution during the past year, find the same occasion as in preceding years for congratulating the patrons and friends of the establishment on its continued and increasing success. They find reasons for such congratulation in the growing patronage and support of the institution, in the enlarged and improved opportunities and means of instruction, in the public confidence as evinced by the concourse of students, and above all, in the efficiency of the School for accomplishing the object and purpose of its formation, as proved by the proficiency of the students themselves. In regard to the last-mentioned point, the first, as it is the most important part of their report, your Council have to state that twelve students, wholly educated in this establishment, having presented themselves for examination before the College of Surgeons, London, and Society of Apothecaries during the year, have obtained their diplomas with credit, and that the only student who offered himself for the degree of Bachelor of Medicine at the New University of London was placed by the Faculty in the first class.

"Your attention must now be called to several new appointments which, on the recommendation of the Lecturers, your Council have been induced to make during the past year, and which will require the approval and confirmation of this general meeting. Mr. Bolton, formerly a distinguished Student of the School, and since then a successful private teacher of medicine, has been appointed to the office of Demonstrator of Anatomy; and Mr. Langston Parker, well known by his zealous prosecution of anatomical and physiological science, and an author of acknowledged merit, has been appointed the Lecturer on Descriptive Anatomy; being part of the course which your indefatigable and talented Honorary Secretary, Mr. Sands Cox, has taught for so long a time with so much honour and ability.—In retiring from this department, which, in addition to his other important engagements, Mr. Sands Cox has filled in so distinguished a manner for a period of fourteen years, and in which capacity he became the founder of the School as at present established, his object has been to devote his time and attention more directly and exclusively to the prosecution of surgery and surgical anatomy—an arrangement which cannot fail to redound to the honour and advantage of the institution.—Mr. Samuel Berry has also been appointed Joint Lecturer on Midwifery; and here your Council must record an act of liberality on the part of his colleagues, the Surgeons of the Town Infirmary, who have courteously permitted the students to enjoy the valuable privilege and important advantage of attending, under his superintendence, the lying-in wards connected with that institution."

The Rev. Dr. Buckland, in a most eloquent address, which occupied upwards of an hour, then moved that the report be accepted. Several appropriate speeches were afterwards made by the Earl of Dartmouth, Lord Lifford, the Rev. J. P. Lee, Drs. Golding Bird, Daubeny, Hodgkin, Foville and Costello, in awarding the Prizes; and a vote of thanks to Professor Dr. Buckland, proposed by Sir J. Mordaunt, Bart. was unanimously carried.

